# Human germ line editing—roles and responsibilities

**DOI:** 10.1007/s13238-015-0224-7

**Published:** 2015-10-28

**Authors:** Jeantine E. Lunshof

**Affiliations:** Department of Genetics, University Medical Centre Groningen, 9713 AV Groningen, The Netherlands; Department of Genetics-Church Lab, Harvard Medical School, Boston, MA 02215 USA

## INTRODUCTION

The mere idea of altering the human germ line has caused excitement as well as fears since decades. The good and, in particular, the dubious motives for such interventions and the anticipated troublesome outcomes have been a recurring theme in literature, art, movies. Until recently, these were merely science fiction scenarios. Yet, considerations about the impact of potential future alteration of the human germ line played a prominent role already in debates ahead of the first clinical trials with somatic gene therapy in 1985 (Anderson, 1985; Fletcher, 1985). Now, three decades later, human germ line modification has become a scientific reality with the experiment on CRISPR/Cas9-mediated gene editing in human-tripronuclear-zygotes by Liang et al., as published in this journal (Liang et al., 2015).

## CURRENT RESEARCH

While studies in animal models, including mammals, have shown that on-target germ line editing is possible, Liang et al. report a long list of obstacles and in particular off-target effects that they encountered in edited human germ line cells. The authors emphasize that any clinical application of CRISPR/Cas9 for human germ line alteration will still need much research before it can go ahead. That is true in any case, but the speed of developments in genome editing is unprecedented and greatly improved methods are available for research already now (Davis et al., 2015; Maruyama et al., 2015; Yang et al., 2014).

Also, Liang et al. used tripronuclear zygotes that are being discarded in IVF-procedures because they lack developmental potential—unless, ‘rescued’ by the removal of one sperm nucleus (Kattera and Chen, 2003). Other researchers have optimized that procedure, suggesting that such repaired zygotes could soon become a more common option in assisted reproduction (Fan et al., 2014). This may raise theoretical questions about developmental potential after enucleation ‘therapy’, and the research use of such abnormal zygotes, that in natural reproduction, however, will perish.

For Liang et al. the use of abnormal fertilized zygotes underscores the absence of any intention to explore further embryonal development *in vitro* or *in vivo*, thereby at least formally avoiding some of the ethics controversies. However, their particular protocol should not distract from the possibility of conducting same experiments on intact diploid zygotes, which in many places is subject to restrictions or is prohibited. In the US there is a difference between federal and privately funded research (Reardon, 2015). Moreover, state regulations differ. The NIH has issued a ban on funding of any human germ line editing research (Statement on NIH funding of research using gene-editing technologies in human embryos, 2015). In Europe regulations differ greatly between countries (EuroStemCell, 2015). Germany, one of the larger European countries with major research resources, is known for its very restrictive Embryo Protection Act (The Embryo Protection Act, 1990). Prohibited is the “improper use of human embryos”, that is use for any purpose other than bringing about a pregnancy. Also, the alteration of human germ line cells is prohibited if intended to be used in human procreation. This would preclude in Germany e.g. any genome editing in sperm cells.

## DEVELOPMENTS AND FUTURE APPLICATIONS

Altogether, it is realistic to consider successful CRISPR/Cas9-mediated gene editing in diploid zygotes followed by normal embryonal and fetal development until term birth will be available in the foreseeable future, as one additional option in assisted reproduction.

A recent development is an application to the UK Human Fertilisation and Embryology Authority (HFEA) for a licence to use germ line genome editing for research into recurrent miscarriage (Cressey et al., 2015).

Current clinical options, for with acceptance differs widely among individuals and societies, range from the use of donor gametes—*in vivo* or *in vitro*—to preimplantation genetic diagnosis with embryo selection and, recently mitochondrial DNA transfer. All these interventions stirred emotional debates when first introduced, but found a place in clinical practice or are expected to do so, as in the case of mitochondrial DNA transfer. Through careful selection for or against traits, all of those methods aim at improving the heritable traits of the future child, in particular by eliminating genetic disease traits. The results of such intentional trait selection at the level of the individual—and possible progeny—are no more than a minor factor in the naturally occurring dynamic changes to human germ lines at the level of human populations that are caused by many different factors. None of these has been disruptive of ‘humankind’ or ‘humanity’.

**Box 1:** Alteration of the human germ line

New germ lines are being created continuously, as with every act of procreation every new child has a novel, unique germ line—the only exception being identical twins. Human germ lines are being altered as they are damaged through environmental and occupational exposures and through our own personal life style choices (Yauk et al., 2015; Fowler et al., 2014). Besides, there is the foreseeable but unavoidable damage—unless rescue protocols can be used—to the germ line as a side effect of medical treatments, in particular though radiation and chemotherapy where an individual trade-off has to be made between harms and benefits. Somatic gene therapy can affect the germ line as an unintended side effect. At the level of populations, environmental pressure can, through positive selection, lead to germ line enhancement in individuals with traits that are not found ‘naturally’ in individuals in other populations, adaptation to living at high-altitude is one well-known example (Valverde et al., 2015). The persistence, in populations exposed to malaria, of sickle-cell polymorphism with heterozygote advantage is another example of adaptation to environmental pressure, while the trait over time disappears when malaria is eradicated (Hedrick, 2012).					

## EMERGING TECHNOLOGIES—THE COME-BACK OF A DEBATE

While the technology is new, the arguments concerning ethical aspects and potential impact on society mirror those of the debates in the eighties and the nineties—with the Human Genome Project in its earliest stages—when somatic gene therapies raised concerns about not-intended germ line alteration as a side effect (Human Genome, 1989; Lunshof and Zimmerli, 1992). Uncertainty about possible harmful outcomes can be assumed to be much greater when the genome alteration is accidental—and harm may go unnoticed for long—than when interventions are targeted and have been tested in model systems, and mathematical predictions can be made. While uncertainty about outcomes can complicate ethical analysis, it is not a moral criterion itself, as sometimes suggested in the recent debate (Lanphier and Urnov, 2015). Harmful consequences can make an act morally objectional, as much as beneficial outcomes can make a course of action recommendable. Uncertainty as such is not a sufficient basis for judgement, it needs further specification.

The still open biological questions concerning human germ line editing can only be resolved through extensive basic research. Clinical trials cannot begin until those questions have been answered, and the burden of responsibility is here on the researchers who are involved in the field. Addressing the ‘societal’ questions can be less easily assigned to specific groups or individuals, as it is not clear who has what role and what responsibilities, moreover, what do we mean when referring to ‘society’? The usual reference to broad societal discussion is well intended and sounds correct, but actually merely adds to vagueness on where decisions are made and by whom.

The ‘uptake’ question is much clearer. Once germ line genome editing becomes available as an option in assisted reproduction, there will be people who want to use it while many others may never opt for this technology.

## FEASIBILITY, USE, AND AVAILABILITY—HUMAN GERM LINE EDITING IN PRACTICE

The roles and responsibilities in human germ line editing can also be outlined by, first, looking at the beginnings, namely at the developers, the scientists who enable the technology and their responsibility for feasibility and safety. Second, by looking at the other end of the development phase, namely the uptake of the technology and the responsibility of the individuals who wish to make use of it.

The actual availability of the technology, however, cannot be described in any clear-cut way, as it will be determined by the laws and regulations of a given country, the international and national guidelines of the professional associations, and the prevailing social and cultural norms in a population.

### Feasibility and biological safety

Technical feasibility and biological safety are preconditions for any next steps towards development and implementation of the technology. It is the role of the scientists to conduct highest quality fundamental research. In genome engineering, the biologists and molecular engineers are those who are responsible in the first place. The work in the lab is subject to biosafety regulations and rules for good laboratory practice (GLP). In addition, national or institutional regulations may apply when work is done using human bodily material. These rules vary among countries, and determine ‘availability’ (see also below). Feasibility and safety are global. While hurdles may be encountered at an early stage, one should keep in mind that these likely will be transient. Therefore, arguments assuming the technology is ultimately “impossible” or “unsafe” may not be sustainable over time. As in other areas, the criteria for good laboratory practice in the work with human reproductive cells and embryos are subject to consensus in the discipline and it is the role of the professional societies to set the standards that guide the research in the field (The ISSCR Statement on Human Germline Modification, 2015). Good practices and safety, however, should not be confused with acceptability of risks—those judgements are ultimately personal, and perceptions differ also among researchers.

### Use

In the end, individuals decide about the use of the technology. Individuals or couples, in their role of prospective parents, decide to use—or not to use—reproductive technologies. As before, their options range from abstaining from having their biologically own children to use of the most advanced assisted procedures, and their prospective parenthood comes with a specific responsibility. This will not be different when germ line genome editing will be among the options. Moreover, the technology must be reliable and safe, meeting the highest standards at the given point in time. Individual choice may be limited by actual availability in the same way as is currently the case with among other options preimplantation genetic diagnosis, gamete donation or surrogacy. As long as there are national or regional differences, people will travel to those places where for them relevant procedures are being offered.

On the other hand, no one should be put under pressure—be it by their family or by their government—to make a particular reproductive choice.

### Availability

While feasibility and safety are universal conditions, and the use of assisted reproduction is based on personal decisions, the actual availability of options is not in the hands of either scientists or propspective parents. The real-world availability of assisted reproductive technologies depends on rules that permit or restrict research activities as well as personal use—and those rules and regulations vary nationally, regionally, locally. In addition, strong moral value-based arguments dominate the debate, which makes universal agreement hard to attain even within countries or among population groups. Yet, in most if not all commentaries, ‘society’ is referred to as the key place of decision-making, often with the ideal of global consensus. That latter goal is vague and unrealistic, and historical attempts at creating rules that should be binding worldwide—as among United Nations member states—have ultimately failed, as recently detailed in an overview by Isasi and Knoppers (Isasi and Knoppers, 2015). On 2 October 2015, UNESCO released the *Report of the IBC on Updating Its Reflection on the Human Genome and Human Rights* addressing recent developments in biomedicine with focus on genetics and genomics, including genome editing (International Bioethics Committee (IBC), 2015). While conservative in tone and calling for restrictions, such a document has no legally binding force.

## PUTTING CONSEQUENCES IN PERSPECTIVE

The potential disruptive effect of human germ line editing seems very small, compared to the potential effects of other applications of high precision genome editing, for example, the use of gene editing that influences ecosystems (Lunshof, 2015). At this moment, the use of CRISPR-based alterations in wild animal populations, in combination with a gene drive, is considered the most impactful application of genome editing. In this scenario, the genome alterations rapidly spread through a wild animal or plant population and the engineered organisms are self-propagating, thereby enabling the reduction or elimination of disease vectors, pests, and invasive species. Unintentional or premature release of engineered organisms from the laboratory can have far-reaching consequences that may affect the environment and human health and well-being (Akbari et al., 2015).

Therefore, the case of genome editing for ecosystem management requires decision-making at an appropriate scale and global deliberations are needed that take into account a diversity of value systems as well as effective modes of governance (Oye et al., 2015).

The case of using CRISPR/Cas9 for human germ line interventions is fundamentally different, as influencing heritable traits in humans will have few consequences beyond the individual, the line of descendants, and their communities. The concrete decision to make use of the technique will ultimately be a personal one, as with any decision concerning reproduction, whether assisted or not.
